# Aldolase A promotes proliferation and G_1_/S transition via the EGFR/MAPK pathway in non-small cell lung cancer

**DOI:** 10.1186/s40880-018-0290-3

**Published:** 2018-05-08

**Authors:** Hailu Fu, Huijun Gao, Xiaoyu Qi, Lei Zhao, Donghua Wu, Yuxin Bai, Huimin Li, Xuan Liu, Jun Hu, Shujuan Shao

**Affiliations:** 1 Liaoning Key Laboratory of Proteomics, Dalian Medical University, No. 9, West Section, South Lvhsun Road, Lvshunkou District, Dalian, 116044 Liaoning P. R. China; 2 Department of Pancreatic and Biliary Surgery, The First Hospital of China Medical University, Shenyang, 110001 Liaoning P. R. China

**Keywords:** ALDOA, NSCLC, Proliferation, G_1_/S, EGFR/MAPK, Cyclin D1, Aerobic glycolysis

## Abstract

**Background:**

Our previous study demonstrated that aldolase A (ALDOA) is overexpressed in clinical human lung squamous cell carcinoma and that ALDOA promotes epithelial–mesenchymal transition and tumorigenesis. The present study aimed to explore the function of ALDOA in the modulation of non-small cell lung cancer (NSCLC) proliferation and cell cycle progression and the potential mechanism.

**Methods:**

ALDOA was knocked down by short hairpin RNA in H520 and H1299 cells. ALDOA was overexpressed with vectors carrying the full-length ALDOA sequence in H1299 and H157 cells. The proliferation capacities were assessed with immunohistochemical staining, Cell Counting Kit-8 and colony formation assays. The cell cycle distribution was examined by flow cytometry, and molecular alterations were determined by western blotting. Cell synchronization was induced with nocodazole. The stability of *cyclin D1* mRNA was tested. The pyruvate kinase M2 and ALDOA protein distributions were examined. Aerobic glycolysis was evaluated with Cell Titer-Glo assay, glucose colorimetric assay and lactate colorimetric assay.

**Results:**

ALDOA knockdown inhibited the proliferation and G_1_/S transition in H520 cells. Conversely, ALDOA overexpression promoted the proliferation and G_1_/S transition in H157 cells. The cell cycle synchronization assay showed that ALDOA expression increased in the G_1_ phase and G_1_/S transition. Furthermore, ALDOA knockdown reduced cyclin D1 expression by regulating epidermal growth factor receptor/mitogen-activated protein kinase (EGFR/MAPK) pathway. Similar results were found in H1299 and H157 cells. The inhibition of mitogen-activated protein kinase kinase 1/2 prompted the nuclear distribution of ALDOA. Additionally, ALDOA knockdown reduced nuclear distribution of PKM2, the extracellular lactate and intracellular adenosine triphosphate concentrations and elevated the extracellular glucose concentration.

**Conclusions:**

ALDOA contributed to activation of the EGFR/MAPK pathway, thus promoting cyclin D1 expression and enhancing proliferation and G_1_/S transition in NSCLC. Additionally, ALDOA facilitated NSCLC aerobic glycolysis.

## Background

Lung cancer is the leading cause of cancer death worldwide, with a morbidity rate of 1.8 million and a mortality rate of 1.6 million persons per year [1, 2]. Non-small cell lung cancer (NSCLC) accounts for up to 80% of all lung cancer cases. Lung adenocarcinoma is the most common subtype, followed by lung squamous cell carcinoma [3]. To some extent, conventional therapies prolong the survival time of patients with early-stage NSCLC [4]. However, the 5-year survival rate for NSCLC patients with metastasis ranges from 4% to 17% [5]. It has been estimated that up to 69% of patients with NSCLC could have a promising molecular target [6]. However, the targeting of irregular signaling has been less efficacious than expected. Therefore, improvements in early diagnosis and molecular-targeted therapy are of great significance for improving the survival rate of patients with NSCLC.

Cell cycle deregulation is a crucial signal that indicates aberrations in cell proliferation. The cell cycle is factitiously divided into four phases: G_1_, S, G_2_ and M [7]. The G_1_, S and G_2_ phases are collectively known as interphase, during which DNA and proteins are synthesized for mitosis [8]. Checkpoints monitor the cell cycle. Once an abnormality is sensed, a set of machineries is initiated until the irregular compartment is recovered or cleared; if abnormalities persist, cells undergo programmed death or transfer to a quiescence status, namely the G_0_ phase [9]. Chemotherapeutic drugs often target cells in a specific phase of the cell cycle [10–12]. Therefore, identification of cell cycle deregulation could provide an early warning of tumor occurrence and a potential approach to cancer treatment.

Aldolase A (ALDOA) is a glycolytic enzyme that contributes to maintenance of the cytoskeleton [13, 14]. ALDOA is widely expressed in almost all tissues and organs, particularly in adult muscle and blood cells [15]. Research has repeatedly shown that ALDOA is overexpressed in various types of cancers including pancreatic cancer [16], hepatocellular carcinoma [17], colorectal cancer [18] and lung cancer [19, 20]. We previously reported that ALDOA is excessively expressed in clinical human lung squamous cell carcinoma compared with adjacent normal tissues and that knockdown of ALDOA impairs the invasion and migration capacities of the lung squamous cell carcinoma cell line H520 [19]. Moreover, the results from a subcutaneous xenograft assay in nude mice implied that ALDOA-knockdown H520 cells possess a weaker capacity for tumorigenesis than cells transfected with negative control short hairpin RNA [19]. However, the underlying mechanism and its potential clinical significance remain unclear.

In this study, we analyzed the effect of ALDOA knockdown on the proliferation and cell cycle progression of NSCLC and further explored the underling mechanisms and potential of ALDOA as a biomarker or drug target for NSCLC.

## Methods

### Cell culture and drug treatment

The human lung squamous cell carcinoma cell line H520 and the human lung adenocarcinoma cell lines H157, A549, H1299 and Anip973 were cultured in RPMI1640 (Gibco, Shanghai, China) supplemented with 10% fetal bovine serum (Gibco) and incubated with 5% CO_2_ at 37 °C. Actinomycin D (Sigma-Aldrich, St. Louis, MO, US) was used to block *cyclin D1* transcription at a dose of 5 μg/mL. The mitogen-activated protein kinase kinase 1/2 (MEK1/2) inhibitor U0126-EtOH (Selleck Chemicals, Houston, TX, US) was used at a dose of 0.5 μmol/L. Epidermal growth factor (EGF) (PeproTech, Rocky Hill, NJ, US) was used at a dose of 50 ng/mL to stimulate the EGF receptor/mitogen-activated protein kinase (EGFR/MAPK) pathway.

### Plasmids and transfection

A pGPU6/GFP/Neo vector carrying short hairpin RNA of ALDOA (shALDOA or shAL) or negative control sequence (shNC) (GenePharma, Suzhou, China) was transfected to H520 cells with Lipofectamine 2000 (Invitrogen, Carlsbad, CA, US). Stably transfected cells were selected by adding 400 μg/mL G418 (Invitrogen) and maintained in 200 μg/mL G418. pcDNA 4.0 vector carrying ALDOA full-length cDNA or control sequence (Abgent, Suzhou, China) was transfected to H157 and H1299 cells. Protein or mRNA was extracted 48–72 h after transfection.

### Xenografts and immunohistochemistry

A subcutaneous tumor formation experiment was performed as described by Du et al. [19]. Dissected xenografts were fixed in 4% paraformaldehyde (PFA) and paraffin-embedded. The slides were de-waxed in xylene and rehydrated in graded alcohol, followed by antigen retrieval in 10 mmol/L sodium citrate buffer. Endogenous peroxidase was inhibited with 1% H_2_O_2_ and washed in phosphate-buffered saline (PBS). Nonspecific binding sites were blocked in goat serum for 30 min at room temperature. The sections were then incubated with rabbit anti-Ki-67 primary antibody (Proteintech, Wuhan, China) and rabbit anti-cyclin D1 primary antibody (Abcam, Cambridge, MA, US) at 4 °C overnight followed by incubation in a biotinylated secondary antibody and peroxidase-labeled streptavidin complex detection (Golden Bridge Biotechnology, Beijing, China). The expression and distribution of Ki-67 (Proteintech) and cyclin D1 (Abcam) were then observed under a microscope (Nikon, Tokyo, Japan).

### Cell Counting Kit-8 (CCK-8) and colony formation assay

Cell viability was tested using CCK-8 (Dojindo Molecular Technologies, Kumamoto, Japan) and colony formation assays. Cells were seeded in a 96-well plate (2000 cells/well). Medium containing 10 μL of CCK-8 reagent and 100 μL of culture medium was added into each well at 0, 24, 48 and 72 h after the cells had become adherent. The cells were incubated for another 2 h, and the absorbance at 450 nm was examined on a microplate reader (Thermo Fisher Scientific, Waltham, MA, US). For the colony formation assays, the cells were plated in a 6-well plate (500 cells/well) for 10 days. The cells were then fixed with 4% PFA (Amresco, Solon, OH, US) and stained with 0.5% crystal violet (Amresco) for 20 min. Colonies of > 50 cells were counted under a light microscope (Olympus, Tokyo, Japan).

### Cell cycle distribution analysis

A cell cycle analysis kit (KeyGen Biotech, Nanjing, China) was used to monitor the cell cycle distribution. Cells under different treatments were harvested and fixed by cold 70% ethyl alcohol overnight at 4 °C, washed twice with PBS, incubated with 100 μL RNase, stained with 100 μg/mL propidium iodide for 30 min on ice, and subjected to flow cytometry analysis (FACS Calibur flow cytometer; BD Biosciences, Franklin Lakes, NJ, US).

### Cell cycle synchronization assay

Nocodazole (0.5 ng/mL; Sigma-Aldrich) was added to synchronize H520 cells into the G_2_/M phase. After 20 h of treatment, the cells were released to the normal cell cycle by adding RPMI1640 medium containing 10% fetal bovine serum. H520 cells were harvested every 2 h, and ALDOA expression was monitored by western blotting. The protein levels of cyclin B1, cyclin D1, cyclin E1 and cyclin A1 were measured to indicate the G_2_/M, G_1_, G_1_/S or S phase of the cell cycle.

### Western blotting

Cells were lysed using radioimmunoprecipitation assay lysis buffer containing 1% phenylmethanesulfonyl fluoride on ice for 30 min, followed by centrifugation at 12,000 rpm at 4 °C. The protein concentration was measured using a bicinchoninic acid assay kit (Beyotime, Shanghai, China). Protein was separated by 8% to 12% sodium dodecyl sulfate polyacrylamide gels and transferred to nitrocellulose membranes (Millipore, Darmstadt, Germany) followed by blocking with 5% nonfat dry milk in PBS containing 0.1% Tween-20. The membranes were incubated with corresponding primary antibody at 4 °C overnight. Primary antibodies for cyclin D1 (1:1000), cyclin E1 (1:500), cyclin-dependent kinase 4 (CDK4, 1:500), CDK6 (1:500), β-catenin (1:800), pyruvate kinase M2 (PKM2, 1:2000), β-actin (1:5000), octamer-binding transcription factor-1 (Oct-1) (1:2000), β-tublin (1:1000) and EGFR (1:1000) were purchased from Proteintech. Primary antibodies for retinoblastoma protein (Rb, 1:1500), phosphorylated EGFR (p-EGFR) (Tyr1068) (1:1000), p-Raf-1 (Ser259) (1:1000) and MEK1/2 (1:10,000) were purchased from Abcam. Primary antibodies for p-Rb (Ser780) (1:1000), Raf-1 (1:1000), p-MEK1/2 (Ser217/221), extracellular signal-regulated kinase 1/2 (ERK1/2, 1:1000), p-ERK1/2 (Thr202/Tyr204) (1:1000) and *p*-β-catenin (Ser675) were purchased from Cell Signaling Technology (Danvers, MA, US). Membranes were incubated with horseradish peroxidase-conjugated goat anti-rabbit or anti-mouse secondary antibody (Abbkine Scientific, Wuhan, China) for 90 min. The signals were detected with a ChemiDoc XRS + system (Bio-Rad, Hercules, CA, US). Protein quantitation was performed using Gel-Pro Analyzer (Media Cybernetics, Rockville, MD, US).

### Real-time quantitative polymerase chain reaction

Total mRNA was extracted using Trizol (Invitrogen) and reverse-transcribed into cDNA using PrimeScript RT Reagent Kit with gDNA Eraser (Takara, Dalian, China). SYBR Premix Ex Taq (Takara) was used for real-time polymerase chain reaction (PCR). The *β*-*actin* mRNA level was used as the internal control. The primer sequences were as follows: *cyclin D1*: forward GCTGCGAAGTGGAAACCATC, reverse CCTCCTTCTGCACACATTTGAA; *β*-*actin*: forward GAATCAATGCAAGTTCGGTTCC, reverse TCATCTCCGCTATTAGCTCCG. Each experiment was repeated more than three times. The relative expression of target genes was calculated using the 2^−ΔΔCT^ method.

### Adenosine triphosphate, glucose and lactate concentration assessment

A Cell Titer-Glo Luminescent Cell Viability Assay (G7570; Promega, Madison, WI, US) was used to measure the intracellular adenosine triphosphate (ATP) concentration. Cells were plated at 5000 per well in a 96-well plate, and the ATP concentration was assessed 10 h later as instructed by the manufacturer. The glucose and lactate concentrations were measured with specific kits (K686, K627; Biovision, Milpitas, CA US). The procedures were performed according to the manufacturer’s online instructions (http://www.biovision.com).

### Immunofluorescence staining

Cells were washed three times with PBS and fixed with 4% PFA for 20 min at room temperature. Cells were penetrated by incubation with 0.3% Triton X-100 (Sigma-Aldrich) for 40 min at room temperature followed by blockage with goat serum (Zsbio, Beijing, China) for 30 min at 37 °C. The cells were then incubated with the corresponding primary antibody diluted in PBS at 4 °C overnight. The secondary antibody conjugated with fluorescein isothiocyanate (Proteintech) was incubated for 90 min at 37 °C followed by staining with 4′,6-diamidino-2-phenylindole (Roche, Basel, Switzerland) at room temperature for 5 min. Images were captured using a Leica microscope (Leica, Wetzlar Germany).

### Statistical analysis

All experiments were performed at least three times. Data were analyzed using SPSS 17.0 software (SPSS Inc., Chicago, IL, US) and are presented as mean ± standard deviation. Student’s *t* test or one-way analysis of variance was used to compare mean differences. A *P* value of < 0.05 was considered statistically significant.

## Results

### ALDOA enhanced the proliferation and G_1_/S transition of NSCLC cells in vitro and in vivo

**Fig. 1:**
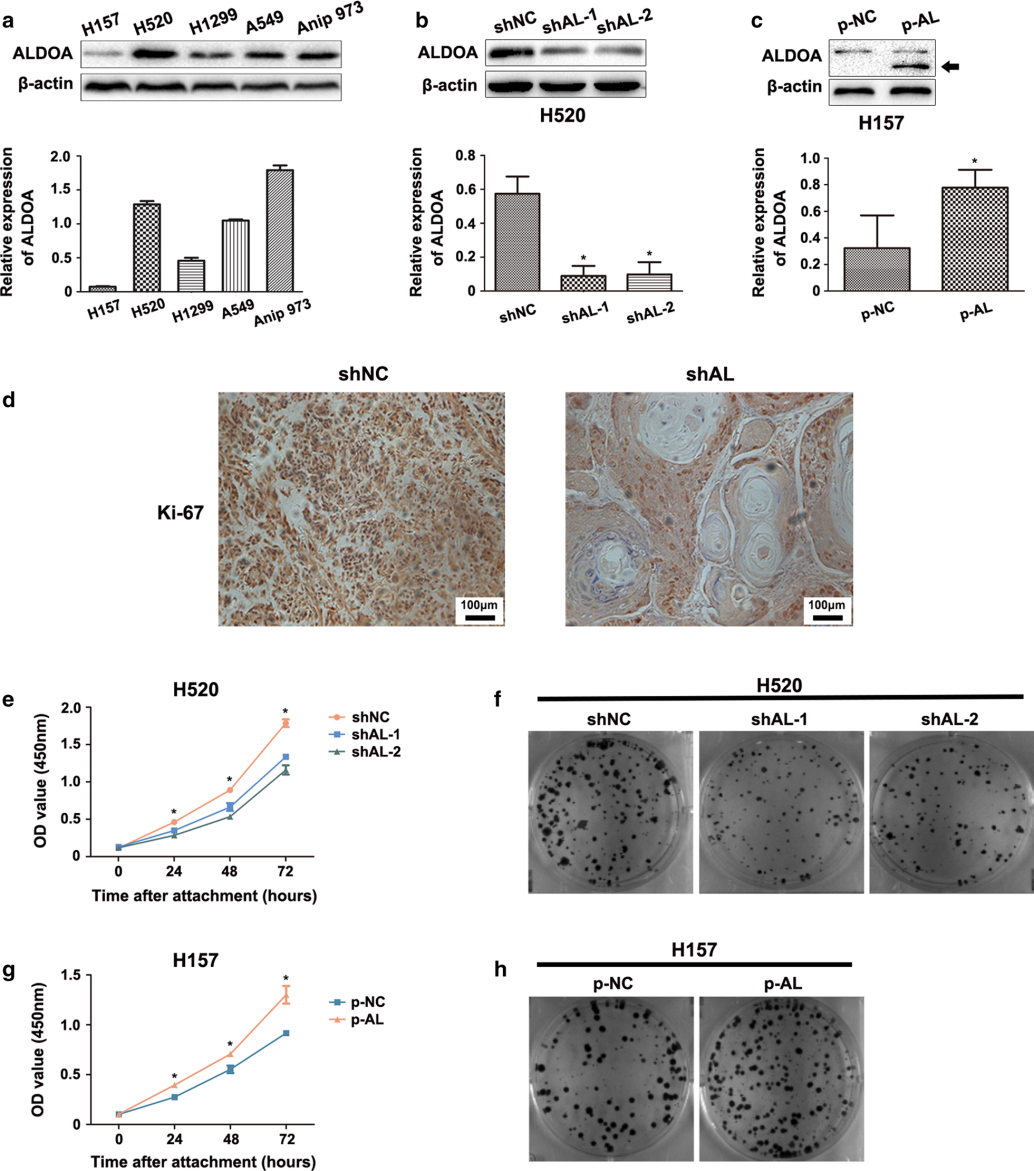
Aldolase A (ALDOA) knockdown decreased the proliferation capacity of non-small cell lung cancer (NSCLC). **a** Comparison of the endogenous ALDOA expression of several NSCLC cell lines. All cell lines except H157 displayed abundant expression of ALDOA. Data are shown as mean ± standard deviation (SD). **b** H520 cells were stably transfected with pGPU6/GFP/Neo-ALDOA-short hairpin RNA (shAL-1, shAL-2) or pGPU6/GFP/Neo-shNC (shNC, negative control). ALDOA was lower in shAL H520 cells than shNC cells. Data are shown as mean ± SD. *P < 0.05 versus shNC. **c** H157 was transfected with pcDNA 4.0 to myc his A vector carrying full-length ALDOA cDNA (p-AL) or control sequence (p-NC). Black arrow indicated exogenous ALDOA. Data are shown as mean ± SD. *P < 0.05 versus shNC. **d** Immunohistochemistry assays for Ki-67 in tumor xenografts. Ki-67 expression was suppressed in shAL H520 cells compared with that in shNC cells. **e** Cell proliferation was assessed by the Cell Counting Kit-8 assay after cells had been attached for 0, 24, 48 and 72 h. The results showed that cell proliferation was inhibited in the shAL group compared with that in the shNC group. Data are shown as mean ± SD. *P < 0.05 versus shNC. **f** Representative images of colony formation assay of the above cells. Cells were fixed and stained with crystal violet 10 days after conventional culture. Both the volume and number of the colonies were lower in the shAL than shNC group. **g** Cell proliferation was promoted in the p-AL group comprared with that in the p-NC group. Data are shown as mean ± SD. *P < 0.05 versus shNC. **h** Representative images of colony formation assay showed that both the volume and number of the colonies were higher in the p-AL than p-NC group

In this study, we first tested ALDOA expression in several NSCLC cell lines. The results demonstrated that all cell lines except H157 displayed abundant expression of ALDOA, and the human lung squamous cell carcinoma cell line H520 presented particularly strong expression (Fig. 1a). Therefore, H520 cell line was utilized as a model in which we knocked down ALDOA to study its function, and H157 was used as a model for ALDOA overexpression. The results indicated that ALDOA protein levels were markedly decreased in H520 cells transfected with shALDOA plasmids (Fig. 1b); an extra band existed in H157 cells transfected with vector carrying full-length ALDOA sequence, which indicated exogenous ALDOA (Fig. 1c). Our prior study indicated that knockdown of ALDOA inhibited H520 cell growth in nude mice [19]. To further confirm this result, we detected Ki-67 expression in these xenografts by immunohistochemistry. Ki-67 is commonly accepted for use as an indicator of the prognosis of various types of cancers and as a marker positively correlated with cancer proliferation [21–23]. The results revealed that Ki-67 expression was suppressed in ALDOA-knockdown xenografts (Fig. 1d). Consistently, in the CCK-8 and colony formation assays, ALDOA-knockdown H520 cells exhibited an attenuated survival capacity (Fig. 1e, f). In contrast, we found that ALDOA overexpression in H157 cells significantly promoted cell proliferation (Fig. 1g, h). These findings indicate that ALDOA contributes to the proliferation of NSCLC both in vitro and in vivo.

**Fig. 2:**
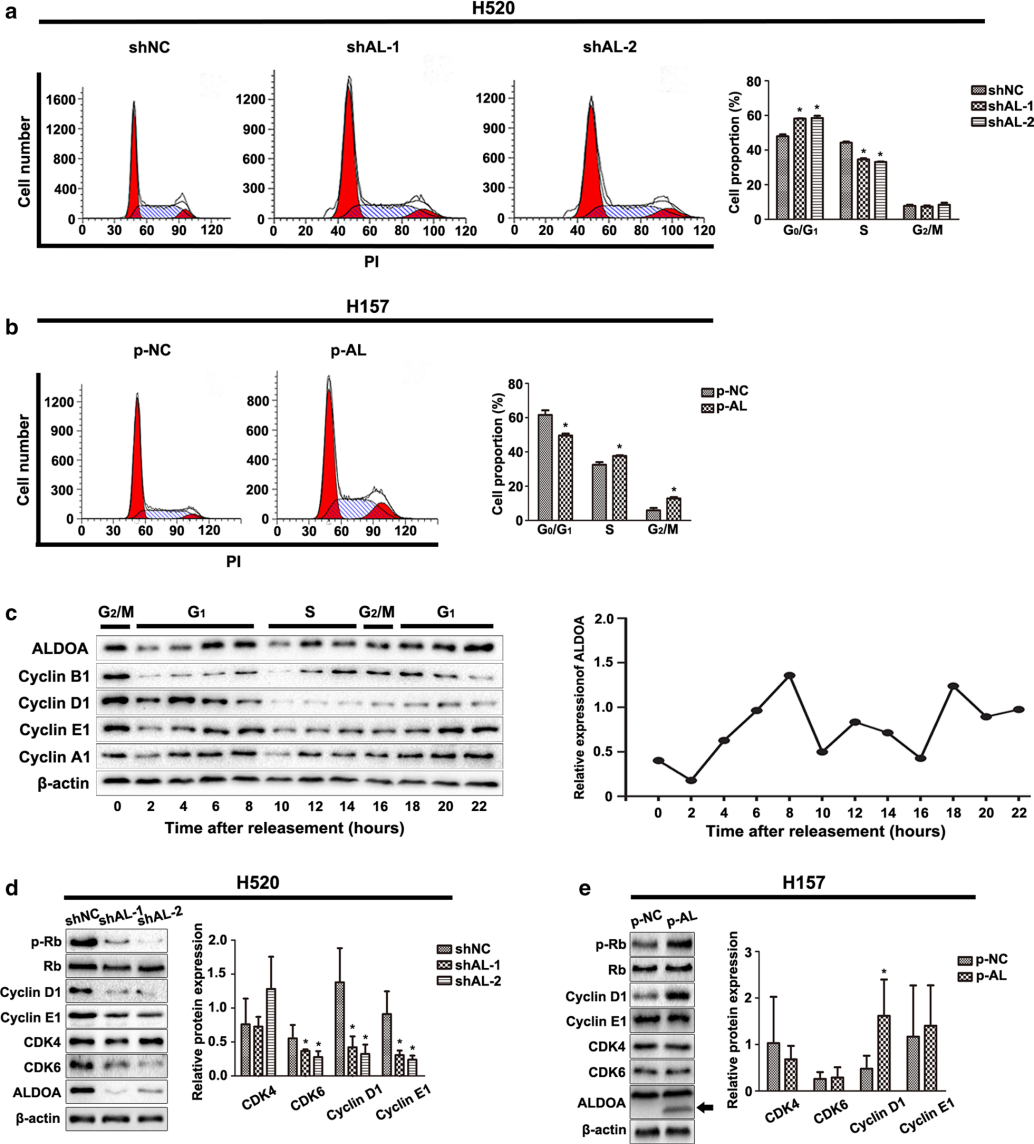
ALDOA contributed to G_1_/S transition in NSCLC. **a** Flow cytometry analysis of H520 cells after propidium iodide (PI) staining. The percentage of cells in the G_0_/G_1_ phase was significantly increased in the shAL group compared with that in shNC group. Data are shown as mean ± standard deviation (SD). *P < 0.05 versus shNC. **b** The percentage of cells in the G_0_/G_1_ phase was significantly decreased in p-AL compared with that in p-NC group. **c** H520 cells were synchronized by the microtubule depolymerizing agent nocodazole for 20 h, released in RPMI1640 supplemented with 10% fetal bovine serum, and harvested every 2 h thereafter. ALDOA was measured by western blotting. Cyclin B1, cyclin D1, cyclin E1 and cyclin A1 were monitored as indicators of the G_2_/M, G_1_, G_1_/S and S phase, respectively. The protein levels were quantified by Gel-Pro Analyzer (Media Cybernetics, Rockville, MD, US). The experiment was repeated three times, and representative data are shown. The results suggested that the protein levels of ALDOA increased in the G_1_ phase and G_1_/S transition. β-actin served as loading control. **d** Retinoblastoma protein (Rb), phosphorylated Rb (p-Rb) (Ser780), cyclin D1, cyclin E1, cyclin-dependent kinase 4 (CDK4) and CDK6 were tested by western blotting. Cyclin D1, cyclin E1, CDK6 and p-Rb (Ser780) were decreased in shAL H520 cells compared with shNC cells, and CDK4 and Rb remained unchanged. β-actin served as loading control. Data are shown as mean ± SD. *P < 0.05 versus shNC. **e** p-Rb and cyclin D1 were increased in p-AL cells compared with that in p-NC cells. Rb, CDK4, CDK6 and cyclin E1 remained unchanged. β-actin served as loading control. Data are shown as mean ± SD. *P < 0.05 versus shNC

We examined the cell cycle distribution to further elucidate the mechanism by which ALDOA regulates NSCLC proliferation. First, we performed flow cytometry assays and found that ALDOA knockdown in H520 cells increased the proportion of cells in the G_0_/G_1_ phase and reduced the proportion of cells in the S phase (Fig. 2a). Correspondingly, ALDOA overexpression in H157 cells decreased the proportion of cells in the G_0_/G_1_ phase and increased the proportion of cells in the S phase and G_2_/M phase (Fig. 2b). Thus, we preliminarily concluded that ALDOA played an important role in sustaining G_1_/S progression. Next, to validate this function, we conducted a cell cycle synchronization assay using the microtubule depolymerizing agent nocodazole [24] to induce mitotic prometaphase arrest and discovered that ALDOA protein expression gradually increased throughout the G_1_ phase and G_1_/S transition (Fig. 2c). This result further supports the notion that ALDOA gives rise to G_1_/S progression.

### ALDOA transcriptionally promoted cyclin D1 expression

The above results demonstrate that ALDOA contributes to cell proliferation probably by modulating G_1_/S transition. G_1_/S transition is precisely orchestrated by CDK4, CDK6 and CDK2 and their corresponding binding activator cyclin D1 and cyclin E1 [25]. Therefore, to define the mechanism by which ALDOA heightens G_1_/S transition, we quantitated the expressions of cyclin D1, cyclin E1, CDK4 and CDK6 by western blotting. Knockdown of ALDOA caused decreases in CDK6, cyclin D1 and cyclin E1 protein levels in H520 cells (Fig. 2d). Moreover, knockdown of ALDOA reduced p-Rb, while total Rb remained unchanged (Fig. 2d). Conversely, the protein levels of cyclin D1 and p-Rb were higher in ALDOA-overexpressed H157 cells than in control cells (Fig. 2e). Rb is a target of CDK4/CDK6 and is phosphorylated once cyclin D1 binds to CDK4/CDK6. p-Rb is then released from E2 promoter binding factor, which in turn acts as a transcription factor that targets several genes engaged in cell cycle progression and cell proliferation, including cyclin E1 [26, 27]. Collectively, these results demonstrate that ALDOA positively regulates cyclin D1 and cyclin E1 expression, accounting for G_1_/S arrest.

**Fig. 3:**
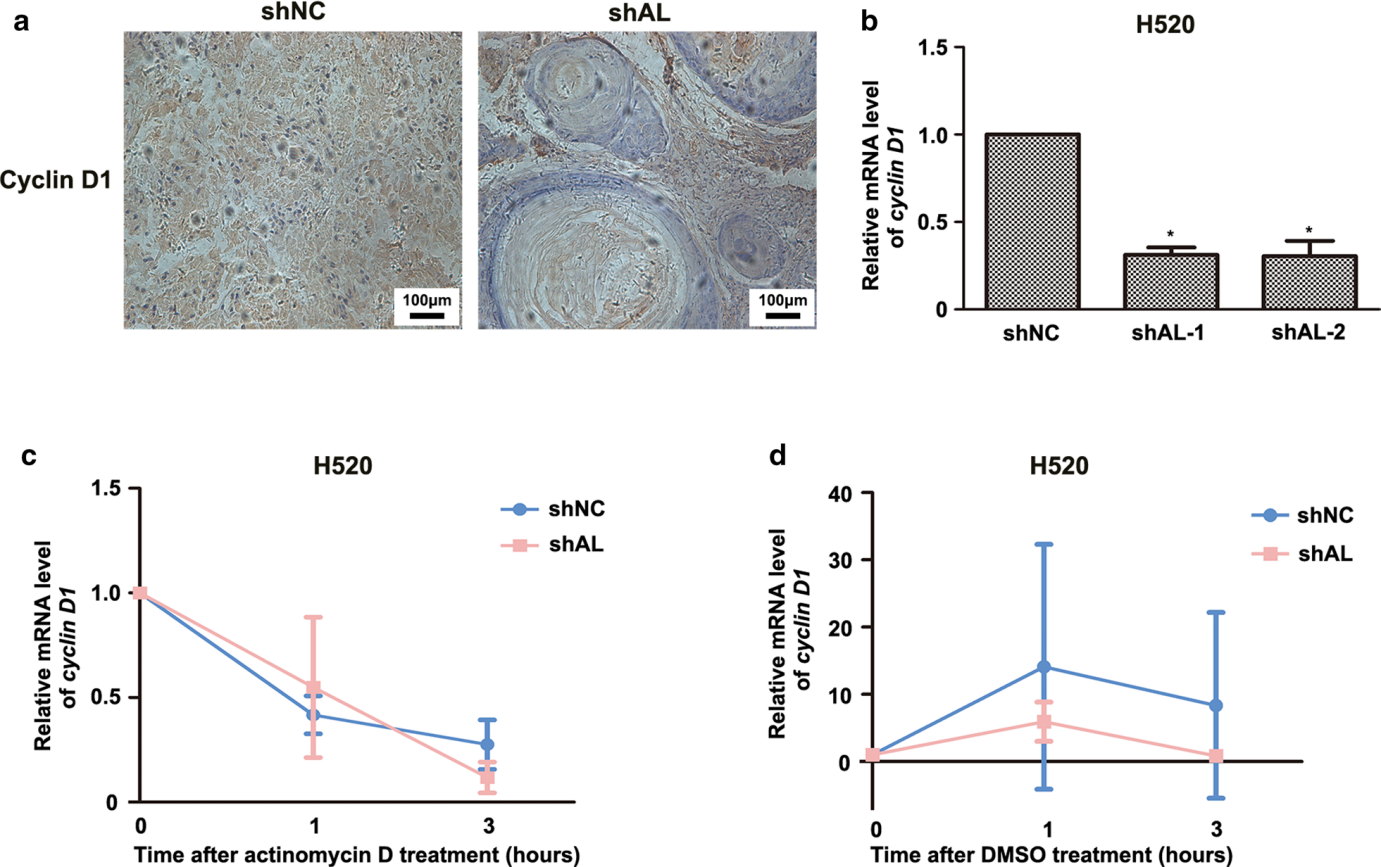
Aldolase A positively regulated cyclin D1 expression at the transcriptional level in H520 cells. **a** Immunohistochemistry assays of cyclin D1 in subcutaneous tumor xenografts of H520 were performed. Cyclin D1 expression was inhibited in the shAL group compared with the shNC group. **b** The mRNA levels of *cyclin D1* in the indicated cells were determined by real-time polymerase chain reaction with *β*-*actin* as an internal control. The mRNA level of *cyclin D1* was lower in the shAL than shNC group. Data are shown as mean ± SD. *P < 0.05 versus shNC. **c** Degradation rates of *cyclin D1* mRNA after transcription inhibition with actinomycin D (0.5 μg/mL) for indicated periods of time. The mRNA half-life of *cyclin D1* showed no significant difference between shAL and shNC cells. Data are shown as mean ± SD. **d** Dimethyl sulfoxide (DMSO) treatment served as control of actinomycin D treatment. The mRNA levels of *cyclin D1* fluctuated in medium supplemented with DMSO

As mentioned above, cyclin E1 expression is indirectly regulated by cyclin D1. Additionally, cyclin D1 showed the most drastic change of all. Therefore, we chose cyclin D1 as the subject for further mechanistic study. Cyclin D1 is the main regulator of CDK4/6 and is upregulated in most lung cancer cells [28–30]. To obtain more convincing results, we also checked cyclin D1 expression by immunohistochemistry in nude mice xenografts. The ALDOA-knockdown group exhibited weaker staining for cyclin D1 than the negative control tissue (Fig. 3a), indicating that ALDOA contributes to cyclin D1 expression in vivo. To assess whether ALDOA regulates *cyclin D1* gene transcription, we tested the mRNA level of *cyclin D1* with real-time PCR and found that *cyclin D1* mRNA was remarkably decreased in ALDOA-knockdown H520 cells (Fig. 3b). Subsequently, to verify whether ALDOA regulates cyclin D1 expression through transcription or mRNA stability, we utilized actinomycin D to inhibit transcription [31] and found that the half-life of *cyclin D1* mRNA was sustained at nearly the same level in ALDOA-knockdown cells as in the control cells (Fig. 3c). Dimethyl sulfoxide was added as a control treatment, and the results suggested that ALDOA fluctuated during the cell cycle without inhibiting transcription (Fig. 3d). These findings collectively indicate that ALDOA regulates cyclin D1 expression at the transcriptional level.

### ALDOA modulated cyclin D1 expression and cell proliferation in an EGFR/MAPK pathway-dependent manner

**Fig. 4:**
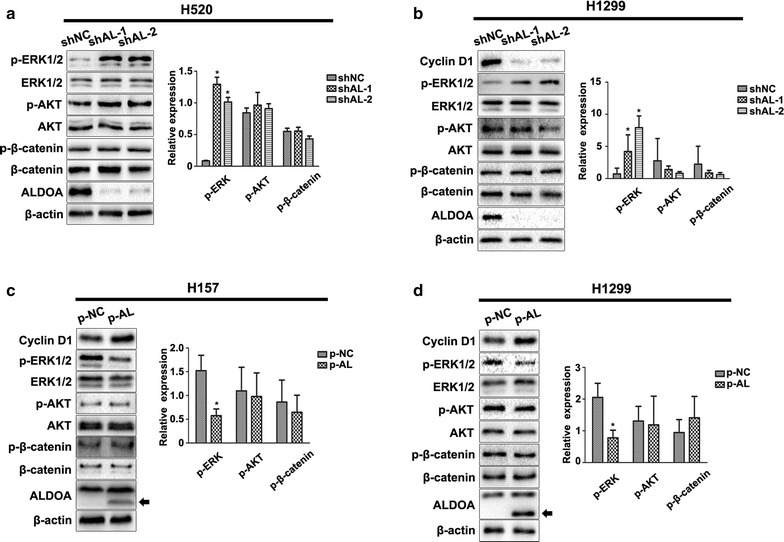
ALDOA inhibited the phosphorylation of extracellular signal-regulated kinase 1/2 (ERK1/2) in NSCLC. Key proteins in the mitogen-activated protein kinase, phosphoinositide-3-kinase/protein kinase B (PI3K/AKT) and Wnt/β-catenin pathways that are involved in *cyclin D1* transcription were evaluated by western blotting. The results revealed that ALDOA knockdown promoted the phosphorylation of ERK1/2 instead of AKT and β-catenin in **a** H520 and **b** H1299 cells. In contrast, ALDOA overexpression inhibited the phosphorylation of ERK1/2 instead of AKT and β-catenin in **c** H157 and **d** H1299 cells. Black arrows indicated exogenous ALDOA. Data are shown as mean ± SD. β-actin served as loading control. *P < 0.05 versus shNC

**Fig. 5:**
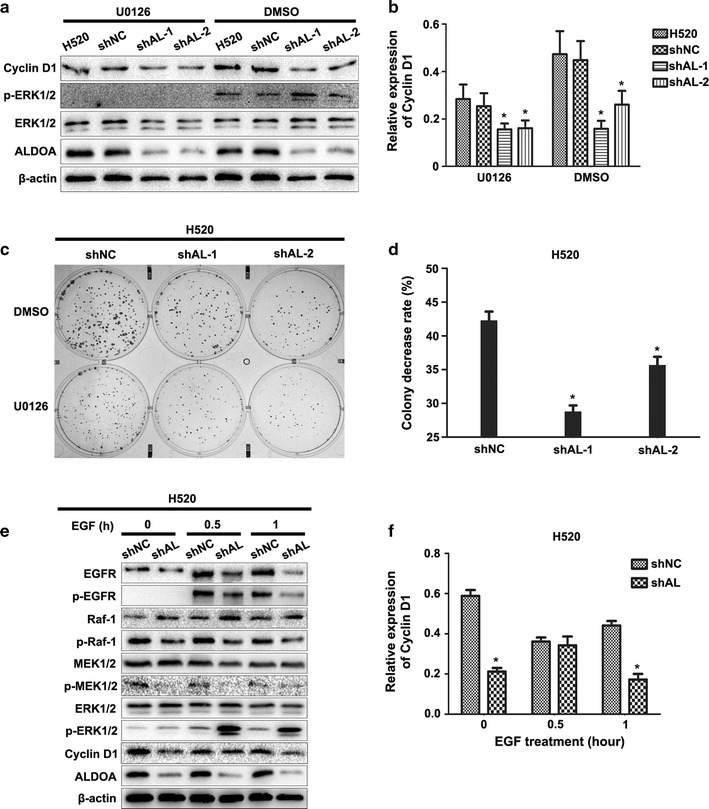
ALDOA enhanced H520 cell proliferation and cyclin D1 expression in an epidermal growth factor receptor/mitogen-activated protein kinase (EGFR/MAPK) pathway-dependent manner. **a** Cyclin D1 expression was tested by western blotting after treatment with mitogen-activated kinase kinase 1/2 (MEK1/2) inhibitor U0126-EtOH or DMSO control treatment. Cyclin D1 protein was quantified by western blotting. Data are shown as mean ± SD in (**b**). β-actin was used as loading control. *P < 0.05 versus shNC. **c** Colony formation assays after MEK1/2 activity blockade with U0126-EtOH. The colony numbers were counted, and the rate of decrease in each group was calculated using the following formula: decrease rate = (colony number of DMSO treatment − colony number of actinomycin D treatment)/colony number of DMSO treatment. The results indicated that rate of decrease in the colony number was higher in the shNC than shAL-1 or shAL-2 group. Data are shown as mean ± SD in (**d**). *P < 0.05 versus shNC. **e** Cells were serum-starved for 24 h or supplemented with 50 ng/mL epidermal growth factor (EGF) for 0.5 or 1 h, respectively. Cyclin D1 and relevant proteins that are engaged in the EGFR/MAPK pathway were measured by western blotting. The results showed that cyclin D1 protein was almost the same in the shAL and shNC group cells with EGF treatment for 0.5 h. Data are shown as mean ± SD in (**f**). β-actin was used as loading control

Pathways that regulate *cyclin D1* transcription include the Wnt/β-catenin, phosphoinositide-3-kinase/protein kinase B (PI3K/AKT) and MEK/ERK pathways [32]. To elucidate the specific mechanism by which ALDOA regulates cyclin D1 expression, we evaluated key proteins that are engaged in these three pathways. Western blotting demonstrated that ALDOA knockdown in H520 and H1299 cells notably enhanced ERK1/2 phosphorylation at Thr202/Tyr204 without impairing total ERK1/2. Concomitantly, AKT, β-catenin and their specific active forms were not obviously altered (Fig. 4a, b). In contrast, ALDOA overexpression in H1299 and H157 cells resulted in a marked reduction of ERK1/2 phosphorylation at Thr202/Tyr204 without impairing AKT or β-catenin activation (Fig. 4c, d). Hence, we postulated that ALDOA contributes to cyclin D1 expression through the MEK/ERK pathway. ERK1/2 belongs to the ERK family and is also termed MAPK; it is a downstream kinase of the EGF pathway. MEK1/2 directly mediates ERK1/2 phosphorylation and thus provokes the binding of ERK1/2 target transcription factors such as activator protein-1, signal transducer and activator of transcription factor 3 (STAT3), and nuclear factor kappa B subunit to genes such as *cyclin D1*, promoting transcription [33]. To confirm this assessment, we administrated U0126-EtOH, which is a MEK1/2-specific inhibitor, and tested cyclin D1 expression and colony formation ability. The results revealed that the effect of ALDOA on the regulation of these processes was almost counteracted following U0126-EtOH exposure (Fig. 5a–d). Moreover, we utilized EGF to stimulate EGFR/MAPK activation [34]. Similarly, activation of EGFR/MAPK signaling abrogated this effect 0.5 h after EGF stimulation. However, except for p-ERK1/2, phosphorylation of EGFR and the downstream members in ALDOA-knockdown cells were strikingly diminished 1 h after EGF treatment compared with those in negative control cells. Meanwhile, cyclin D1 expression differed between shAL and shNC cells (Fig. 5e, f). Taken together, these observations indicate that the EGFR/MAPK pathway is a prerequisite for the promotion of cyclin D1 expression and proliferation by ALDOA in NSCLC.

### Detection of a binary regulation loop between ALDOA and the EGFR/MAPK pathway

**Fig. 6:**
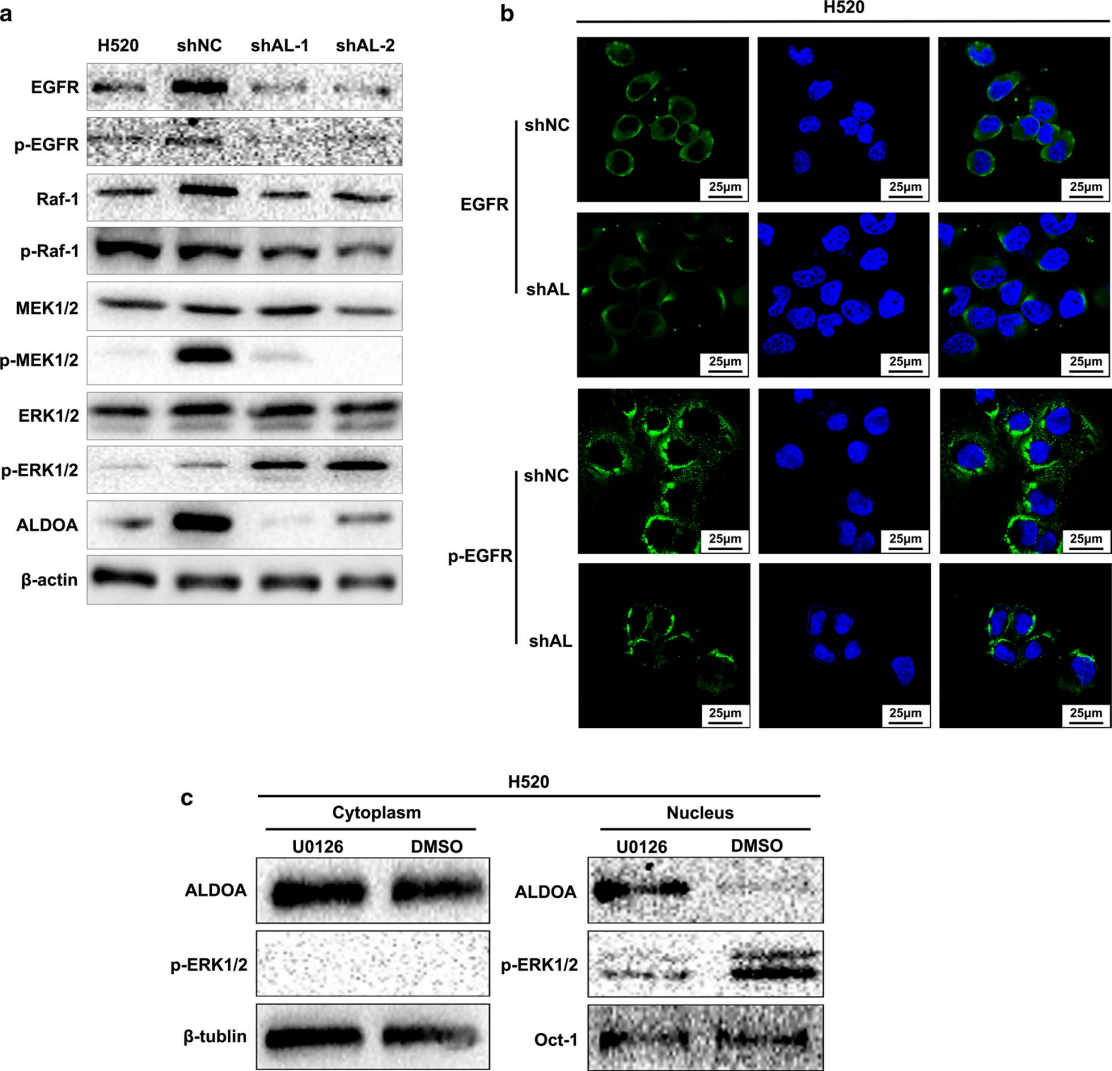
Mutual regulation relationship between ALDOA and epidermal growth factor receptor/mitogen-activated protein kinase (EGFR/MAPK) pathway. **a** Key components of the EGFR/MAPK pathway were measured by western blotting. β-actin was used as loading control. The results showed that EGFR, p-EGFR (Tyr1068), Raf-1, p-Raf-1 (Ser259) and p-MEK1/2 (Ser217/S221) were decreased and that p-ERK1/2 (Thr202/Tyr204) was upregulated in shAL cells compared with shNC cells. **b** Immunofluorescence assays of EGFR and p-EGFR (Tyr1068). EGFR or p-EGFR (Tyr1068) was labeled with fluorescein isothiocyanate-conjugated antibody (green), and the nucleus was labeled by 4′,6-diamidino-2-phenylindole (blue). ALDOA-knockdown H520 cells exhibited weaker staining of EGFR and p-EGFR (Tyr1068) compared with negative control cells. **c** ALDOA distribution after MEK1/2 inhibition. Nuclear and cytoplasmic proteins of H520 cells were separately extracted after U0126-EtOH treatment. The ALDOA and p-ERK1/2 distributions were examined by western blotting. Octamer-binding transcription factor-1 (Oct-1) or β-tublin was used as a loading control of the nucleus or the cytoplasm

The above results provide a preliminary clue that the EGFR/MAPK pathway plays a crucial role in the association between ALDOA and NSCLC proliferation. Activation of the EGFR/MAPK pathway requires multiple steps involving Ras, Raf, MEK, ERK and other components [33, 35]. Western blotting results revealed that EGFR, Raf-1 and their corresponding phosphorylated forms were remarkably reduced in ALDOA-knockdown cells. However, the total MEK1/2 and ERK1/2 protein levels remained the same, p-MEK1/2 (Ser217/221) was decreased, and p-ERK1/2 (Thr202/Tyr204) was elevated (Fig. 6a). Confocal microscopy analysis suggested a similar result in that ALDOA-knockdown H520 cells exhibited weaker staining of EGFR and p-EGFR (Tyr1068) compared with control cells (Fig. 6b). These results indicated that ALDOA knockdown inhibited EGFR activation, but stimulated ERK1/2 activation. Collectively, these findings suggest that ALDOA contributes to triggering the EGFR/MAPK pathway but exerts distinct roles at different levels of this pathway.

It is well accepted that mutual modulation relationships or feedback regulation always exist among biological molecules. Here, we examined the distribution of ALDOA after U0126-EtOH treatment and observed that U0126-EtOH administration led to the nuclear translocation of ALDOA (Fig. 6c). Thus, based on the above results, we conclude that ALDOA and the EGFR/MAPK pathway are mutually regulated.

### ALDOA facilitated aerobic glycolysis of NSCLC

**Fig. 7:**
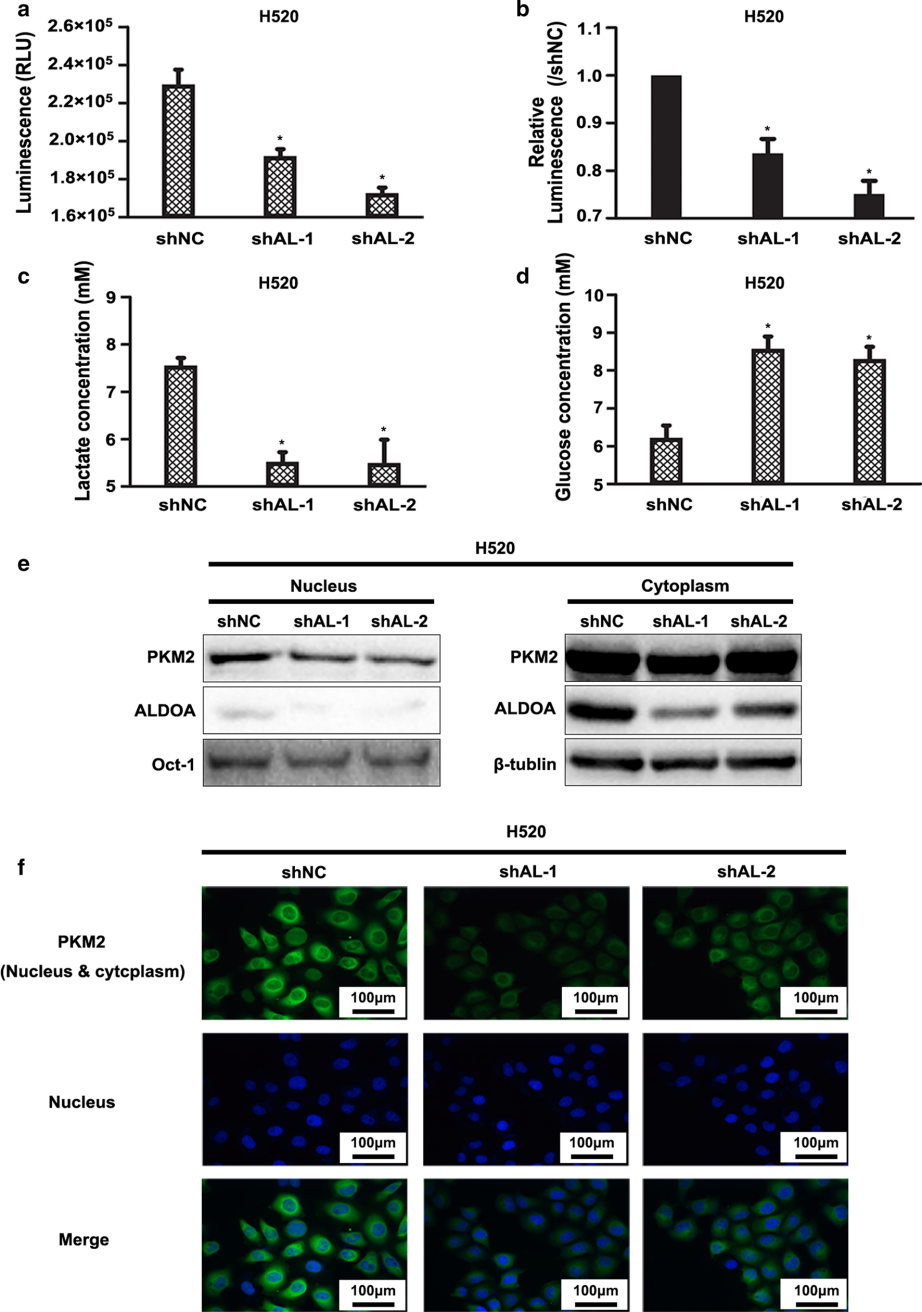
ALDOA-knockdown suppressed aerobic glycolysis of H520 cells. **a** Intracellular adenosine triphosphate (ATP) levels. The Cell Titer-Glo Luminescent Assay Kit was used to test the intracellular ATP concentration in the indicated cell lines. The luminescence signal was measured to reflect the ATP concentration. **b** Luminescence signal of ATP compared with control group (shNC). The results indicated that the intracellular ATP concentration was lower in the shAL than shNC group. *P < 0.05 versus shNC. **c** Extracellular lactate concentration and **d** glucose concentration were measured using lactate and glucose colorimetric assay kits, respectively. Absorbance at 450 nm was measured and then transformed according to the manufacturer to reflect the lactate or glucose concentration of the supernatant. The results suggested that the extracellular lactate concentration was lower and that the extracellular glucose concentration was higher in the shAL than shNC group. *P < 0.05 versus shNC. **e** The pyruvate kinase M2 (PKM2) distribution was determined by western blotting. Nuclear and cytoplasmic proteins were separately extracted. Oct-1 or β-tubulin was used as a loading control of the nucleus and cytoplasm, respectively. **f** Immunofluorescence assay of PKM2. PKM2 protein was labeled with fluorescein isothiocyanate-conjugated antibody (green). The nucleus was stained with 4′,6-diamidino-2-phenylindole (blue). The results in **e** and **f** suggested that PKM2 was predominantly decreased in the nucleus in ALDOA-knockdown H520 cells compared with negative control cells

Enhanced glycolysis or aerobic glycolysis refers to the phenomenon by which cancer cells preferentially depend on glycolysis for energy consumption regardless of oxygen status [36, 37]. Aerobic glycolysis is also recognized as a hallmark of cancer [38]. A decreased ATP concentration, glucose consumption and lactate secretion are crucial indexes used for estimation of decreased glycolysis efficiency [39–41]. As mentioned above, ALDOA is a glycolytic enzyme that catalyzes the reversible conversion of fructose 1,6-bisphosphate to glyceraldehyde 3-phosphate and dihydroxyacetone phosphate; therefore, we assessed these indexes to evaluate whether ALDOA silencing utilizes NSCLC glycolysis. As expected, ALDOA knockdown led to reductions in the intracellular ATP concentration (Fig. 7a, b) and extracellular lactate concentration (Fig. 7c) together with an elevation in the glucose concentration of the supernatant (Fig. 7d). We also examined the distribution of the rate-limiting enzyme PKM2. Nuclear PKM2 is also an important kinase that participates in the proliferation, migration and invasion of many types of cancers [42–45]. Western blotting and immunofluorescence assay showed that the level of nuclear PKM2 was significantly lower in ALDOA-knockdown cells than in control cells, whereas cytoplasmic PKM2 was maintained at almost the same level (Fig. 7e, f). These results suggest that ALDOA plays a role in maintaining NSCLC aerobic glycolysis and PKM2 nuclear location.

## Discussion

In this study, we found that ALDOA promoted the proliferation and G_1_/S transition in NSCLC by upregulating *cyclin D1* transcription in an EGFR/MAPK pathway-dependent manner. We also demonstrated that ALDOA contributed to aerobic glycolysis and nuclear translocation of PKM2.

Lung cancer is lethal partially because of its insidious onset. For most patients, by the time a definitive diagnosis of lung cancer is made, the tumor has already progressed to the metastasis stage and caused functional or structural damage [46]. Uncontrolled cell cycle progression is an early sign of malignant transformation. In the present study, we found that ALDOA knockdown resulted in G_1_/S blockage. Similarly, previous bioinformatics analysis results suggest a correlation between ALDOA and the cell cycle in NSCLC [47]. These results imply that ALDOA plays a role in the occurrence of NSCLC and is a potential early marker of NSCLC.

Accumulating evidence indicates that ALDOA contributes to the proliferation of cancer cells [48–50]. In addition to defining the relationship between ALDOA and the proliferation capacity of NSCLC, we found that the EGFR/MAPK pathway is involved in this regulation. EGFR, also termed HER-1 and ErbB-1, is excessively expressed in 85% of NSCLC cells and is associated with a poor prognosis [51]. Overexpression of EGFR leads to EGFR dimerization, auto-phosphorylation and downstream activation of the PI3 K, STAT and MAPK pathways. Consequently, these pathways mediate the transcription of genes that contribute to transformation, proliferation, angiogenesis, migration, adhesion and invasion [52]. Tyrosine kinase inhibitors targeting EGFR are frequently used in patients with NSCLC [53–55]. These treatments generate certain effects that promote the survival of patients with NSCLC. However, most patients with NSCLC inevitably develop resistance to these drugs, leading to the recurrence and progression of NSCLC [56–58]. Another EGFR-targeting strategy is the use of EGFR monoclonal antibody, which is often used in combination with a tyrosine kinase inhibitor (TKI) or chemotherapy and exhibits certain benefits [59–61]. A recent finding suggests that EGFR gene amplification or protein overexpression confers acquired drug resistance to these EGFR TKIs [62]. This finding indicates that precluding EGFR protein expression is crucial for overcoming acquired EGFR TKI resistance. Our study showed that ALDOA inhibited the expression and activation of EGFR in NSCLC. Consequently, it is plausible that the combined administration of TKI and ALDOA inhibitors might provide efficient treatment for patients with NSCLC with acquired TKI resistance. Additionally, it has been suggested that combined treatment with the EGFR TKI inhibitor rociletinib and EGFR monoclonal antibody cetuximab is effective against acquired TKI resistance in NSCLC [62]. However, the accompanying side effects have not yet been examined, and whether an ALDOA inhibitor could serve as a substitute for cetuximab remains unclear. Further experimental and clinical studies are needed to examine these issues.

Our findings also revealed that ALDOA knockdown attenuated aerobic glycolysis in NSCLC cells. Cancer cells favor aerobic glycolysis to benefit malignancy by decreasing the microenvironmental pH [63, 64], disturbing reactive oxygen species homeostasis [65, 66], regulating chromatin remodeling and contributing to biosynthesis [39–41, 67, 68]. Therefore, knock down of ALDOA also help to relieve stress and cut off the energy supply of NSCLC.

Another interesting result is that ALDOA knockdown increased the phosphorylation of ERK1/2 at Thr202/Tyr204, whereas the corresponding target (cyclin D1) and the phosphorylation of the upstream kinase MEK1/2 were suppressed. This finding is controversial but noteworthy. However, we could not define the exact mechanism by which ALDOA regulates the EGFR/MAPK pathway. We hypothesize that this phenomenon might be explained in the following aspects. First, ALDOA was transferred to the nucleus after MEK1/2 inhibition by U0126-EtOH; similarly, ALDOA has been reported to translocate to the nucleus under various conditions that influence cell proliferation [49]. This finding suggests that ALDOA has the capacity to affect gene transcription. Second, enhanced ERK1/2 phosphorylation does not always facilitate proliferation. There is an evidence that suggests that the marked over-activity of ERK2 phosphorylation attenuates cell proliferation under specific conditions [69]. Moreover, through its effects on aerobic glycolysis, ALDOA is able to alter the microenvironment of cancer and thus impair the regulation of *cyclin D1* transcription.

## Conclusions

ALDOA was involved in EGFR/MAPK pathway activation and contributed to aerobic glycolysis of NSCLC, thus promoting cyclin D1 expression, G_1_/S transition and cell proliferation.

## Abbreviations

NSCLC: non-small cell lung cancer
ALDOA: aldolase A
MAPK: mitogen-activated protein kinase
PCR: polymerase chain reaction
ATP: adenosine triphosphate
PFA: paraformaldehyde
PBS: phosphate-buffered saline
CDK: cyclin-dependent kinase
Rb: retinoblastoma protein
PI3K: phosphatidylinositol 3 kinase
MEK: mitogen-activated protein kinase kinase
ERK: extracellular signal-regulated protein kinase
EGF: epidermal growth factor
EGFR: epidermal growth factor receptor
PKM2: pyruvate kinase M2
DAPI: 4′,6-diamidino-2-phenylindole
AKT: also PKB, protein kinase B
STAT3: signal transducer and activator of transcription factor 3
TKI: tyrosine kinase inhibitor
CCK-8: Cell Counting Kit-8
